# Estimating the probability of multiple incidences of the same cancer type in a single workplace

**DOI:** 10.1093/joccuh/uiae072

**Published:** 2024-11-29

**Authors:** Sintaroo Watanabe, Kota Fukai, Masayuki Tatemichi

**Affiliations:** Safety & Health Group, Japan Marine United Corporation Kure Shipyard, Kure, 737-0027 Japan; Department of Preventive Medicine, Tokai University School of Medicine, Isehara, 259-1193 Japan; Department of Preventive Medicine, Tokai University School of Medicine, Isehara, 259-1193 Japan

**Keywords:** occupational cancer, standardized incidence ratios, cancer epidemiology, chemical exposure

## Abstract

**Objectives:**

This study aimed to estimate the probability of observing 2 cases of the same cancer type in a workplace with 300 employees, to help investigation of occupational cancer.

**Methods:**

We assumed a workplace where chemicals are handled, employing 300 males aged 15 to 64, with an age distribution standardized to Japan’s population from 2016 to 2019. Using national cancer statistics for newly diagnosed cases among males in this age range and period, we calculated the expected number of cancer cases for the workplace over a 1- and 10-year period. We computed standardized incidence ratios (SIRs) for 2 instances of the same cancer type within these time frames and estimated the time required for the SIR to reach 2.0 and its lower 95% CI limit (LL) to reach 1.0.

**Results:**

The SIR for any cancer type exceeded 10 and was significantly high when 2 cases occurred within 1 year. Over 10 years, the SIR remained significantly high in some cancer types. The observation periods required for SIR to reach 2.0 and LL to reach 1.0 for any cancer type were at least 5.4 and at least 1.7 years, respectively.

**Conclusions:**

Considering that over 99% of Japanese workplaces employ fewer than 300 people, the likelihood of observing the same cancer type within 1 year is low. This study enhances our understanding of occupational cancer incidence and supports the integration of such data into prevention strategies.

## Key points

**What is already known on this topic:** Previous epidemiological studies have played an important role in identifying occupational cancers linked to chemical exposures. However, it is unclear how to determine whether each type of cancer has a higher incidence based on the size of workplace.

**What this study adds:** This study simulates the occurrence of the same cancer at specific frequencies in a workplace and compares these incidences with those in the general population. By calculating the ratio of incidence rates for each type of cancer, it provides guidelines for determining whether a cancer is occurring abnormally in the workforce.

**How this study might affect research, practice, or policy:** If multiple cases of the same type of cancer arise in a workplace, this study offers a method to promptly assess whether the cancer incidence is high. This will help to investigate occupational cancer, and enable early intervention and the implementation of measures to prevent subsequent occurrences of occupational cancer.

## 1. Introduction

Occupational cancers remain a significant concern, evidenced by the reports of multiple cases within specific industries. Notable examples include bile duct cancer in workers exposed to 1,2-dichloropropane (1,2-DCP) and dichloromethane (DCM) at an offset color-proof printing factory^1^ and bladder cancer in workers exposed to orthotoluidine^2^ and 3,3′-dichloro-4,4′-diaminodiphenylmethane (MOCA) in dye factories.^3^ These cancers have been linked to occupational factors based on epidemiological studies. Furthermore, handling chemicals has been associated with an increased risk of lung, pancreatic, and bladder cancers, even when no specific causative agents have been identified.^4^ Long-term chemical handling in workplaces has also been linked to increased incidence of lung, oropharyngeal, pancreatic, and bladder cancers.^5^

The International Agency for Research on Cancer (IARC) underscores the crucial role of epidemiological studies in assessing the carcinogenic risks of chemicals. Notably, 1,2-DCP and DCM were reclassified from “not classifiable as to its carcinogenicity to humans (Group 3)” and “possibly carcinogenic to humans (Group 2B)” to “carcinogenic to humans (Group 1)” and “probably carcinogenic to humans (Group 2A),” respectively, following the identification of cancer clusters among workers.^6^

Despite these advancements, the extent of risk that workers face regarding chemical-induced occupational cancers, including the frequency and types of cancers, remains unclear. The first step is to evaluate whether cancer incidence is higher in chemical-exposed workers when investigating chemical-induced occupational cancer. Then, it is crucial for timely occupational health interventions and prevention of subsequent cases. This study aimed to simulate expected cancer occurrences in a single workplace that handles chemicals and is of a certain size. For this research, we set the workplace size at approximately 300 employees because over 99% of Japan’s 5.91 million workplaces employ fewer than 300 people.^7^ The goal was to estimate the epidemiological probability of observing the same type of cancer within the workplace over specific time frames.

## 2. Materials and methods

In this simulation study, we compared the incidence of cancer in the general male population with that of a group of male workers, aged 15-64 years, who were assumed to be exposed to chemicals in Japan from 2016 to 2019. We limited the simulation to males because the handling of chemicals in the workplace is primarily performed by male workers due to legal regulations on certain chemicals, and there are significant natural gender differences in cancer incidence rates (IRs) among the working-age population.^4^^,^^5^ Primary outcomes were the incidence rate ratios (IRRs) and age-standardized incidence ratios (SIRs), considering the age structure of Japanese companies,^8^ as well as their 95% CIs, by cancer types. Secondary outcomes were expressed as years needed to reach 2.0 for IRRs or SIRs, and years needed to reach 1.0 for their lower 95% CI limit (LL). This method was the same as that used to investigate occupational bladder cancer by Ward et al^9^ and occupational bile duct cancer by Kumagai et al.^1^

This simulation study was solely based on published data; hence, materials and ethical approval were not required.

### 2.1. Reference group

The National Cancer Center Japan provided data on newly diagnosed cancer cases by type, sex, and 5-year age groups from 2016 to 2019.^10^ The cancer IR for males aged 15-64 years was calculated and designated as the cancer incidence for the reference group. To minimize annual variability in cancer IR, data from the 4 years 2016 to 2019 were aggregated.

For the reference group, let PY be the total person-years for this population, calculated as the sum of annual values from 2016 to 2019. Let 𝑎 be the number of new cancer cases for each cancer type diagnosed during these 4 years. The cancer IR in the general male population can be calculated as follows:


(1) \begin{align*} \mathrm{IR}=\frac{a}{\mathrm{PY}} \end{align*}

We calculated person-years by multiplying the number of the population in each age group by the number of years, based on the demographic statistics.^8^ Herein, PY was 153 302 000 person-years (Table S1).

### 2.2. Assumptions for chemical exposure group

For a cohort of 𝑁 male workers aged 15-64 years handling chemical substances, followed for 𝑥 years, let *b* be the number of workers newly diagnosed with cancer during this period. The IR for *x* years (${\mathrm{IR}}_{b\mid x}$) of cancer in the chemical workers’ cohort was calculated as follows:


(2) \begin{align*} {\mathrm{IR}}_{b\mid x}=\frac{b}{N\times x} \end{align*}

### 2.3. Outcome and measures

#### 2.3.1. IRR

The numbers of person-years of observation and cancer incidence were determined and defined as $\mathrm{PY}$ and $a$, respectively. The term $a$ was different for each cancer type, and $\mathrm{PY}$ was 153 302 000 person-years (Table S1). The number of new cancers expected to be diagnosed during *x* years (Ex*_x_*) for *N* workers is expressed by the following equation:


(3) \begin{align*} {\mathrm{Ex}}_x=\frac{N\times x\times a}{\mathrm{PY}} \end{align*}

The IRR comparing the cancer IRs between chemical workers and the reference group was defined as ${\mathrm{IRR}}_{b\mid x}$ and calculated as follows:


(4) \begin{align*} {\mathrm{IR}\mathrm{R}}_{b\mid x}=\frac{{\mathrm{IR}}_{b\mid x}}{\mathrm{IR}}=\frac{b}{{\mathrm{Ex}}_x} \end{align*}

The years *x* needed to reach ${\mathrm{IRR}}_{b\mid x}$ was determined by the following equation:


(5) \begin{align*} \mathrm{Years}\ x\ \mathrm{needed}\ \mathrm{to}\ \mathrm{reach}\ {\mathrm{IRR}}_{b\mid x}=\frac{b}{N\times \mathrm{IR}\times{\mathrm{IRR}}_{b\mid x}} \end{align*}

#### 2.3.2. SIR

Owing to the differences in age structure between the general population and employees in small and medium-sized enterprises with fewer than 300 workers, we used an age-standardization method to calculate the SIRs as follows. To calculate the SIR, we first determined the expected number of cases by multiplying the age-specific population by the age-specific cancer incidence rates of the reference population. The SIR was then calculated by comparing this expected number with the actual observed cancer cases.

Let ${p}_i$ represent the proportion of male workers in each age group, and ${a}_i$ and ${\mathrm{PY}}_i$ represent the number of cancer cases and person-years of observation for each age group in the reference population, respectively. Taking into account the age structure of *N* workers, the expected number of cancer cases after $x$ years of observation, denoted as ${\mathrm{Ex}}_{sx}$, is generally calculated using the following equation. The detailed calculations are provided in Table S2:


(6) \begin{align*} {\mathrm{Ex}}_{sx}=N\times \left(\sum \frac{p_i\times{a}_i}{{\mathrm{PY}}_i}\right)\times x \end{align*}

The subscript *s* indicates standardized. The SIR when *b* new cancer cases occur for workers who handle chemicals in *x* years $\big({\mathrm{SIR}}_{b\mid x}$) is expressed by the following equation:


(7) \begin{align*} {\mathrm{SIR}}_{b\mid x}=\frac{b}{{\mathrm{Ex}}_{sx}} \end{align*}

The years *x* needed to reach ${\mathrm{SIR}}_{b\mid x}$ was determined by the following equation:


(8) \begin{align*} \mathrm{Years}\ x\ \mathrm{needed}\ \mathrm{to}\ \mathrm{reach}\ {\mathrm{SIR}}_{b\mid x}=\frac{b}{N\times \left(\sum \frac{p_i\times{a}_i}{{\mathrm{PY}}_i}\right)\times{\mathrm{SIR}}_{b\mid x}} \end{align*}

We referred to the national statistics to determine the proportion by age group of males aged 15-64 employed in companies with fewer than 300 employees.^8^ Taking the actual age distribution into account, substituting 300 for *N* results in Table S1.

#### 2.3.3. Simulation in a single workplace with 300 male employees

This simulation was conducted for a workplace involved in handling chemicals, based on the following 4 assumptions: (1) the workplace employs 300 male workers who handle chemicals; (2) the number of workers at the workplace has remained constant over time; (3) workers are continuously employed from the start of their employment until they are diagnosed with cancer; and (4) the association between chemical exposure and cancer is investigated once 2 cases of the same type of cancer have been diagnosed among the workers.

The assumption of a workplace with 300 workers handling chemical substances was based on the fact that workplaces with fewer than 300 workers account for 99.7% of all workplaces in Japan.^7^ This assumption accurately reflects the situation in the majority of Japanese workplaces. Signs of high cancer incidence in chemical-exposed workers need to be promptly detected to implement effective occupational health measures. Therefore, we assumed that an investigation should be conducted when 2 cases of the same type of cancer occur. In this simulation, we substituted *N* = 300 and *b* = 2 in eqns (2) to (8). We set a cutoff value for the IRR and SIR at ≥2.0 and their LL at ≥1.0 to determine that cancer incidence is high in chemical-exposed workers. In Japan, regulations for occupational cancer prevention require reporting when more than 1 case of the same type of cancer arises within a workplace within a 1-year period. This threshold is consistent with the reasoning used in US courts, where a relative risk greater than 2.0 has been deemed sufficient to infer that exposure to a particular agent more likely than not caused the disease.^11^ Such a 2-fold increase in risk has been recognized as indicative of a causal relationship between exposure and disease. Collectively, we adopted LL for IRR or SIR = 1.0 as a cutoff value. In this simulation study, we expect the CIs to be wider because we assumed a small number of cancer cases, namely 2. Even in this situation, this indicates that the increase of cancer incidence is beyond the level that can be explained by chance.

We assumed that 2 out of 300 male workers exposed to chemicals developed the same cancer, and the following calculations were performed: IRR and SIR for 2 cases of the same cancer in a 1-year period (IRR_2|1_, SIR_2|1_), IRR and SIR for 2 cases of the same cancer in a 10-year period (IRR_2|10_, SIR_2|10_), and the years needed to reach IRR and SIR = 2.0 and their LL = 1.0.

For sensitivity analysis, we calculated IRR_2|1_ and determined the years needed to reach IRR = 2.0 and LL = 1.0, assuming a scenario where all employees in a 300-person workplace were elderly, aged between 60 and 64 years. Calculations of eqns (1) to (8) were performed using Microsoft Excel 2019 (Microsoft Corporation, WA, USA). The 95% CIs for IRR and SIR were obtained by substituting the numbers in eqns (4) and (7) using the Standardized Mortality Ratio calculation function in the Open Epi Version 3.01 (www.openepi.com). Years needed to reach LL for IRR or SIR = 1.0 are in principle equal to the value of the LL of IRR_2|1_ or SIR_2|1_.

## 3. Results

The cancer incidence in Japanese males aged 15-64 years from 2016 to 2019 and the estimated IRRs for 2 cases of the same cancer type in a single workplace with 300 male employees are shown in Table 1. The IRs, which were crude estimates derived from the population structure of Japan at that period, were highest for colorectal cancer, followed by stomach, lung, prostate, and kidney/urinary tract cancers. In the analysis, assuming a workplace with 300 male employees, the probability of observing 2 cases of the same cancer within 1 year was lowest for colorectal cancer but still showed a significant increase (IRR_2|1_ = 11.7; 95% CI, 2.0-38.6]). The mean IRR_2|1_ exceeded 100 for bladder, skin, gallbladder, bile duct, and other cancers. Even within an observing period of 10 years, the probability of observing 2 cases of the same cancer (IRR_2|10_) showed a significant increase for pancreatic cancer, leukemia, thyroid gland cancer, bladder cancer, skin cancer, gallbladder/bile duct cancer, central nervous system cancer, larynx cancer, and multiple myeloma. The years needed to reach an IRR of 2.0 were 5.8 years for colorectal cancer, 8.7 years for stomach cancer, 10.0 years for lung cancer, followed by 21.7 years for kidney/urinary tract cancer, and 50.9 years for bladder cancer, indicating a considerable duration for all cases. The years needed to reach LL for IRR of 1.0 were 2.0 years for colorectal cancer, 2.9 years for stomach cancer, 3.3 years for lung cancer, followed by 7.3 years for kidney/urinary tract cancer, and 17.1 years for bladder cancer, indicating a considerable duration for all cases. The years needed to reach the LL for IRRs of 1.0 were fewer than those needed for IRRs of 2.0. The IRRs in this case were 6.0.

**Table 1:** Cancer incidence in Japanese males aged 15-64 years from 2016 to 2019 and the estimated incidence rate ratios observing 2 cases of the same cancer type in a single workplace with 300 male employees.

**Cancer type**	**Reference group** ^**a**^		**Workplace with 300 male employees**			
	**Incident cases (*n*)**	**Incidence rate (/100 000 population)**	**1-year IRR (95% CI)**	**10-year IRR (95% CI)**	**Years needed to reach IRR = 2.0**	**Years needed to reach LL of IRR = 1.0** ^**b**^
	**𝑎**	**IR: (𝑎/PY) × 100 000**	**IRR**_**2|1**_ **: IR**_**2|1**_ **/IR**	**IRR**_**2|10**_ **: IR**_**2|10**_ **/IR**	**2/(300 × IR × 2.0)**	
**Colorectal**	87 595	57.1	11.7 (2.0-38.6)	1.0 (0.2-3.9)	5.8	2.0
**Stomach**	59 060	38.5	17.3 (2.9-57.2)	1.7 (0.3-5.7)	8.7	2.9
**Lung**	51 285	33.5	19.9 (3.3-65.8)	2.0 (0.3-6.6)	10.0	3.3
**Prostate**	44 112	28.8	23.2 (3.9-76.6)	2.3 (0.4-7.7)	11.6	3.9
**Kidney/urinary tract**	23 509	15.3	43.5 (7.3-143.6)	4.3 (0.7-14.4)	21.7	7.3
**Oral/pharyngeal**	20 505	13.4	49.8 (8.4-164.7)	5.0 (0.8-16.5)	24.9	8.4
**Malignant lymphoma**	20 316	13.3	50.3 (8.4-166.2)	5.0 (0.8-16.6)	25.2	8.4
**Liver**	19 298	12.6	53.0 (8.9-175.0)	5.3 (0.9-17.5)	26.5	8.9
**Esophagus**	18 766	12.2	54.5 (9.1-179.9)	5.4 (0.9-18.0)	27.2	9.1
**Pancreas**	17 075	11.1	59.9 (10.0-197.8)	6.0 (1.0-19.8)	29.9	10.0
**Leukemia**	11 259	7.3	90.8 (15.2-299.9)	9.1 (1.5-30.0)	45.4	15.2
**Thyroid gland**	10 237	6.7	99.8 (16.7-329.8)	10.0 (1.7-33.0)	49.9	16.7
**Bladder**	10 048	6.6	101.7 (17.1-336.0)	10.2 (1.7-33.6)	50.9	17.1
**Skin**	8832	5.8	115.7 (19.4-382.3)	11.6 (1.9-38.2)	57.9	19.4
**Gallbladder/bile duct**	5630	3.7	181.5 (30.4-599.8)	18.2 (3.0-60.0)	90.8	30.4
**Central nervous system**	5517	3.6	185.2 (31.1-612.0)	18.5 (3.1-61.2)	92.6	31.1
**Larynx**	3636	2.4	281.1 (47.1-928.7)	28.1 (4.7-92.9)	140.5	47.1
**Multiple myeloma**	3237	2.1	315.7 (52.9-1043.0)	31.6 (5.3-104.3)	157.9	52.9

Abbreviations: IR, incidence rate; IR_2|1_, IR of 2 cases in 1 year per 300 population (=2/300); IR_2|10_, IR of 2 cases in 10 years per 300 population (=2/300 × 10); IRR, incidence rate ratio, LL, lower 95% CI limit; PY, person-years (=153 302 000).

a Statistics from the national cancer registry from 2016 to 2019 in Japan.

b Same value for LL of IRR_2|1_ in principle. IRRs for each cancer type were 6.0 in this case.

SIRs based on the population structure of workplaces with <300 employees are presented in Table 2. The rank order of SIR magnitude remained the same for the IRR, even after age adjustment. The SIRs were 6.3%-12.1% lower than the IRRs. SIR_2|1_ (the SIR of observing 2 cases of the same cancer within 1 year) was the lowest for colorectal cancer and showed a significant increase (SIR_2|1_ = 10.2; 95% CI, 1.7-33.7). The mean SIR_2|1_ exceeded 100 for skin, gallbladder/bile duct, and other cancers. Within an observing period of 10 years, SIR_2|10_ showed a significant increase for leukemia, thyroid gland cancer, bladder cancer, skin cancer, gallbladder/bile duct cancer, central nervous system cancer, larynx cancer, and multiple myeloma. The years needed to reach an SIR of 2.0 were >5 years for all cancer types and >10 years for cancers other than colorectal, stomach, and lung cancers. The years needed to reach the LL for an SIR of 1.0 were >1.7 years for all cancer types and >5 years for cancers other than colorectal, stomach, lung, and prostate cancers. The years needed to reach the LL for SIRs of 1.0 were fewer than those needed for SIRs of 2.0. The SIRs in this case were 6.0.

**Table 2:** Age-standardized incidence ratios observing 2 cases of the same cancer type in a single workplace with 300 male employees.

**Cancer type**	**1-year period**		**10-year period**			
	**Expected cases**	**SIR (95% CI) observing 2 cases**	**Expected cases**	**SIR (95% CI) observing 2 cases**	**Years needed to reach SIR = 2.0**	**Years needed to reach LL of SIR = 1.0** ^**a**^
	**Ex**_***s*1**_	**SIR**_**2|1**_ **: 2/Ex**_***s*1**_	**Ex**_***s*10**_	**SIR**_**2|10**_ **: 2/Ex**_***s*10**_	**2/(Ex**_***s*1**_ **× 2.0)**	
**Colorectal**	0.19	10.3 (1.7-34.0)	1.94	1.0 (0.2-3.4)	5.2	1.7
**Stomach**	0.13	15.4 (2.6-50.8)	1.30	1.5 (0.3-5.1)	7.7	2.6
**Lung**	0.11	17.7 (3.0-58.4)	1.13	1.8 (0.3-5.8)	8.8	3.0
**Prostate**	0.10	20.7 (3.5-68.5)	0.96	2.1 (0.3-6.9)	10.4	3.5
**Kidney/urinary tract**	0.05	38.2 (6.4-126.3)	0.52	3.8 (0.6-12.6)	19.1	6.4
**Oral/pharyngeal**	0.04	44.7 (7.5-147.5)	0.45	4.5 (0.7-14.8)	22.3	7.5
**Malignant lymphoma**	0.04	45.1 (7.6-149.1)	0.44	4.5 (0.8-14.9)	22.6	7.6
**Liver**	0.04	46.9 (7.9-155.1)	0.43	4.7 (0.8-15.5)	23.5	7.9
**Esophagus**	0.04	48.3 (8.1-159.4)	0.41	4.8 (0.8-15.9)	24.1	8.1
**Pancreas**	0.04	52.8 (8.9-174.5)	0.38	5.3 (0.9-17.5)	26.4	8.9
**Leukemia**	0.02	85.1 (14.3-281.0)	0.24	8.5 (1.4-28.1)	42.5	14.3
**Thyroid gland**	0.02	90.3 (15.1-298.3)	0.22	9.0 (1.5-29.8)	45.1	15.1
**Bladder**	0.02	90.1 (15.1-297.7)	0.22	9.0 (1.5-29.8)	45.1	15.1
**Skin**	0.02	103.1 (17.3-340.7)	0.19	10.3 (1.7-34.1)	51.6	17.3
**Gallbladder/bile duct**	0.01	164.0 (27.5-542.0)	0.12	16.4 (2.8-54.2)	82.0	27.5
**Central nervous system**	0.01	170.6 (28.6-563.8)	0.12	17.1 (2.9-56.4)	85.3	28.6
**Larynx**	0.01	249.0 (41.7-822.6)	0.08	24.9 (4.2-82.3)	124.5	41.7
**Multiple myeloma**	0.01	277.5 (46.5-916.8)	0.07	27.8 (4.7-91.7)	138.8	46.5

Abbreviations: Ex_*s*1,_ expected newly diagnosed cancer cases in 1 year; Ex_*s*10,_ expected newly diagnosed cancer cases in 10 years; LL, lower 95% CI limit; SIR, standardized incidence ratio.

a Same value for LL of SIR_2|1_ in principle. SIRs for each cancer type were 6.0 in this case.

The results of the sensitivity analysis, which simulated a scenario where all employees were elderly, aged between 60 and 64 years, are presented in Table S3. Even in a workplace consisting of older workers, observing 2 or more cases within 1 year in a cohort of 300 individuals showed a significant increase in IRR for cancers other than colorectal, stomach, prostate, and lung cancer. The years needed to reach an IRR of 2.0 were 2.0 years for lung cancer and 10.3 years for bladder cancer.

## 4. Discussion

The results of this study revealed the rarity of observing multiple cases of the same type of cancer within a short period in workplaces with 300 employees. For colorectal cancer, which has the highest IRs, the SIR exceeded 10.0 and its LL was over 1.0 if 2 cases occurred within 1 year. Moreover, it would take approximately 10 years for the expected number of cases to reach 2 in a population of 300 males. For cancers other than colorectal, stomach, and lung cancers, it takes more than 10 years to exceed an SIR of 2.0. Furthermore, except for colorectal, stomach, lung, and prostate cancers, the LLs for each SIR exceed 1.0 if 2 cases of cancer occur within 5 years, exceeding the level that occurs by chance. Additionally, over 99% of workplaces in Japan employ fewer than 300 employees.^7^ Therefore, in most of the workplaces, if 2 cases of the same type of cancer occur within 1 year in the same workplace, it can be determined that the probability of cancer is increasing. Even when assuming a cohort consisting solely of elderly workers, where cancer IRs are generally higher, observing multiple cases of the same type of cancer remained a rare event for most cancer types. Furthermore, even over a long period of 10 years, the probabilities of incidence were increased for some cancer types.

Several studies have explored the relationship between occupational exposure and cancer, using the general population as a control group. However, to the best of our knowledge, only 4 studies^1^^,^^3^^,^^12^^,^^13^ have investigated exposure groups with <300 individuals, as considered in this simulation study. The SIRs or Standardized Mortality Ratios (SMRs) reported in these studies, in descending order, were as follows: an SMR of 2900 for bile duct cancer associated with exposure to 1,2-DCP and DCM^1^; an SIR of 35-90 for bladder cancer linked to exposure to 4-chloro-*o*-toluidine^12^; an SIR of 3.3 related to exposure to 4,4′-methylene-bis-ortho-chloroaniline^3^; and an SIR of 2.9 associated with work at secondary aluminum smelters.^13^ The LLs for each SIR or SMR were higher than 1.0 except for one study.^3^ Our findings indicate that the rarity of IRRs or SIRs exceeding 2.0 and their LLs exceeding 1.0 in workplaces of this scale aligns with previous research outcomes.

It is also essential to consider the intervals between occurrences when examining cases of occupational cancers. Fukai et al^4^^,^^5^ demonstrated that the incidence of lung, esophageal, pancreatic, and bladder cancers increases with the handling of certain chemicals. However, our study indicates that detecting clusters of these cancer types within workplaces, as considered in this study, would require at least 3.0-15.2 years when adjusted for age structure. For example, Popp et al^12^ retrospectively observed 49 workers exposed to 4-chloro-*o*-toluidine for an average of 18 years and identified 7 cases of bladder cancer. Dost et al^3^ examined a cohort of 308 workers with a history of handling 4,4′-methylene-bis-ortho-chloroaniline over 25 years and identified 2 cases of bladder cancer. Maltseva et al^13^ observed 98 aluminum smelter workers for approximately 50 years and identified 8 cases of bladder cancer. Focusing on the intervals between occurrences of the same type of cancer is crucial. Observing multiple clusters within a 10-year period in workplaces of this scale is a first step in suspecting the influence of chemical exposure.

The methodology of this study can be adapted to the case of bile duct cancer reported by Kumagai et al.^1^ The interval between the first 2 bile duct cancers among the 62 male workers exposed to 1,2-DCP or DCM was 2 or 3 years, respectively. In this case, the IRR was 439.2 (95% CI: 73.6-1451.0) or 292.8 (95% CI: 49.1-967.3), respectively, providing sufficient evidence for increased bile duct cancer in workers exposed to 1,2-DCP or DCM once the first 2 patients had developed bile duct cancer.

Notably, studies focusing on a single workplace are now sparse, with recent research being conducted at national levels, and some even at international levels. A fundamental requirement for such studies is a database that links chemical exposure histories with cancer incidence data. Establishing such a database would facilitate epidemiological research spanning from 10 years to over half a century. Companies responsible for workers’ safety and health cannot easily track the health information of retired workers. In Japan, the Inpatient Clinico-Occupational Database of the Rosai Hospital Group (ICOD-R) is managed by the Japan Organization of Occupational Health and Safety (JOHAS).^4^^,^^5^ However, individuals who develop cancer after chemical exposure do not necessarily visit hospitals affiliated with the JOHAS; hence, it is crucial to have a system that can link occupational history with cancer registration. Furthermore, to effectively transfer the results of epidemiological studies into chemical safety measures, it is crucial to gather detailed exposure information, including the types of chemicals used, the quantities involved, and the management measures under which they were handled. In this regard, the mandatory chemical risk assessment records in Japan are indispensable.^14^

This simulation study offers advantages to occupational health practitioners. First, it provides guidance to occupational health physicians on the relationships between cancer occurrence and chemical exposure in the workplace. In Japan, since April 2023, occupational health physicians are required to assess the association between chemical exposure and cancer when multiple cases of the same cancer occur at workplaces that manufacture or handle chemical substances. Second, by incorporating both IRR and SIR, this study provides a comprehensive analysis that not only reflects the crude cancer risk but also adjusts for age-related confounding, which is particularly important in small- and medium-sized enterprises where the workforce tends to have a higher proportion of older workers. We presented a scenario with 2 cancer cases among men aged 15-64 at a workplace with 300 employees. By entering the workplace size, age structure, observation period, and number of cancer cases into eqns (1) to (8) in the Methods section, occupational health physicians can perform customized analyses tailored to their specific workplaces. We assumed an aging workforce and demonstrated a biased scenario as a sensitivity analysis. Notably, these analyses can be conducted using commonly available software such as Excel and freely available statistical tools.

This study had several limitations. First, inferring a causal relationship between chemical exposure and cancer requires additional evidence beyond cancer incidence probabilities. Although calculating these probabilities is a crucial step, further investigation is needed to consider factors such as chemical management practices in the workplace and other potential risk factors among cancer patients, including genetic predispositions and lifestyle habits. Second, the validity of the reference group may be problematic, as the carcinogenic risk of widely used chemicals may be underestimated if the reference group includes individuals exposed to these chemicals. This highlights the need for more refined reference groups in future studies. Third, setting an SIR of 2.0 as the threshold for precautionary measures may have been too high. Although this standard is based on court findings of a causal relationship between drug or chemical exposure and disease causation, recent classifications by the IARC, such as welding fumes being deemed carcinogenic with a reported 1.4-fold lung cancer risk, suggest that lower thresholds might be more appropriate. Additionally, some recent epidemiological studies using SIR as an outcome have indicated a potential association between occupation and cancer with SIRs as low as 1.2.^15^ In this simulation, the IRR or SIR was greater than 2.0 for significantly higher cancer incidence because this study assumed a small number of cancer cases, namely, 2 in a single workplace. However, when a national-scale data aggregation system is established and put into operation, it is expected that a significantly higher incidence of cancers will be observed even if the SIR is smaller than 2.0. Further discussion is needed to determine the appropriate SIR value for initiating strategies for preventing occupational cancer.

In conclusion, this study introduced a method to evaluate cancer risk among chemical-exposed workers by simulating cancer occurrences in the workplace and comparing them with rates in the general population. Despite the small number of cases, the significantly elevated SIR for 2 observed cancers suggests the need for investigation. Although this analysis highlights the importance of monitoring cancer incidences in smaller workplaces, additional research is needed to account for factors such as genetic predispositions and lifestyle habits to more accurately determine whether the cancers were caused by chemical exposure. Our findings highlight the rarity of multiple cancer cases in small workplaces, urging vigilance for occupational exposures when clusters occur. Establishing comprehensive databases linking exposure histories with cancer data is crucial for long-term research and precise risk assessments.

## Supplementary Material

Web_Material_uiae072

## Data Availability

The data used in this study are available in the public domain as cited.
