# Supplementary material for: Estimating the probability of multiple incidences of the same cancer type in a single workplace
Source: J Occup Health. 2024 Nov 29;66(1):uiae072. doi: 10.1093/joccuh/uiae072 (PMC11662353; doi:10.1093/joccuh/uiae072)
Supplement: Web_Material_uiae072 [file web_material_uiae072.zip › JOHsw_supplementary_final_2024-11-21.docx]

Supplementary Table 1. Expected number of cancers within a year, adjusted for age structure of workers in companies under 300 male employees.

| Age group (years) | Cancer cases from 2016 to 2019 by cancer type | Total population during 2016 to 2019 | Proportion of males working in workplaces under 300 employees | Expected number of cancers when 300 workers are followed for one year* |
| --- | --- | --- | --- | --- |
| 15-24 | 𝑎_(15-24)_ | 25,129,000 | 8.3% | 300×8.3%×(𝑎_(15-24)_ /25,129,000) ×1 |
| 25-34 | 𝑎_(25-34)_ | 27,181,000 | 17.2% | 300×17.2%×(𝑎_(25-34)_ /27,181,000) ×1 |
| 35-44 | 𝑎_(35-44)_ | 34,573,000 | 23.0% | 300×23.0%×(𝑎_(35-44)_ /34,573,000) ×1 |
| 45-54 | 𝑎_(45-54)_ | 35,880,000 | 29.5% | 300×29.5%×(𝑎_(45-54)_ /35,880,000) ×1 |
| 55-64 | 𝑎_(55-64)_ | 30,539,000 | 21.9% | 300×21.9%×(𝑎_(55-64)_ /30,539,000) ×1 |
| Sum of columns | *a* | 153,302,000(=PY) | 100.0% | $\mathrm{Ex}_{s1}$ |

*Expected number of occurrences over a 10-year period ($\mathrm{Ex}_{s10}$) is equal to 10 times of Ex*_s_*_1_(Ex*_s_*_10_ =10×Ex*_s_*_1_).

Subscript “*s*” in Ex*_s_*_1_ and Ex*_s_*_10_ indicates standardized by age structure of workplace under 300 employees.

Supplementary Table 2. General formula for calculating expected number of cancer cases standardized by age structure.

| Age group (years) | Cancer cases from 2016 to 2019 by cancer type | Total population during 2016 to 2019 | Proportion of males working in workplaces | Expected number of cancers when *N* workers are followed for *x* years |
| --- | --- | --- | --- | --- |
| 15-24 | 𝑎_(15-24)_ | PY_(15-24)_ | $p$_(15-24)_ | *N*×$p$_(15-24)_×(𝑎_(15-24)_/PY_(15-24)_)×*x* |
| 25-34 | 𝑎_(25-34)_ | PY_(25-34)_ | $p$_(25-34)_ | *N*×$p$_(25-34)_×(𝑎_(25-34)_/PY_(25-34)_)×*x* |
| 35-44 | 𝑎_(35-44)_ | PY_(35-44)_ | $p$_(35-44)_ | *N*×$p$_(35-44)_×(𝑎_(35-44)_/PY_(35-44)_)×*x* |
| 45-54 | 𝑎_(45-54)_ | PY_(45-54)_ | $p$_(45-54)_ | *N*×$p$_(45-54)_×(𝑎_(45-54)_/PY_(45-54)_)×*x* |
| 55-64 | 𝑎_(55-64)_ | PY_(55-64)_ | $p$_(55-64)_ | *N*×$p$_(55-64)_×(𝑎_(55-64)_/PY_(55-64)_)×*x* |
| Sum of columns | *a* | PY | 100.0% | $N\times\left( \sum\frac{p_{i}\times a_{i}}{\mathrm{PY}_{i}} \right)\times x=\mathrm{Ex}_{sx}$ |

Abbreviations: PY: person-years.

Subscript “*s*” in Ex*_sx_* indicates standardized by age structure of assumed workplace.

Supplementary Table 3. Estimated incidence rate ratios observing two cases of the same cancer type in a single workplace with 300 male employees all aged 60-64

| Cancer type | Workplace with 300 male employees all aged 60-64 | |  |
| --- | --- | --- | --- |
|  | 1-year IRR (95% CI) | Years needed to reach IRR=2.0 | Years needed to reach LL of IRR=1.0^*a^ |
|  | IRR_2\|1_: IR_2\|1_/IR | 2/(300×IR×2.0) |  |
| Colorectal | 2.9 (0.5–9.6) | 1.4 | 0.5 |
| Stomach | 3.5 (0.6–11.5) | 1.7 | 0.6 |
| Prostate | 3.7 (0.6–12.1) | 1.8 | 0.6 |
| Lung | 4.0 (0.7–13.2) | 2.0 | 0.7 |
| Esophagus | 10.7 (1.8–35.4) | 5.4 | 1.8 |
| Liver | 11.2 (1.9–37.1) | 5.6 | 1.9 |
| Kidney/Urinary tract | 12.4 (2.1–41.0) | 6.2 | 2.1 |
| Oral/Pharyngeal | 13.7 (2.3–45.4) | 6.9 | 2.3 |
| Pancreas | 13.8 (2.3–45.7) | 6.9 | 2.3 |
| Malignant lymphoma | 15.2 (2.6–50.4) | 7.6 | 2.6 |
| Bladder | 20.7 (3.5–68.4) | 10.3 | 3.5 |
| Skin | 36.2 (6.1–119.7) | 18.1 | 6.1 |
| Gallbladder/Bile duct | 36.9 (6.2–121.8) | 18.4 | 6.2 |
| Leukemia | 39.4 (6.6–130.1) | 19.7 | 6.6 |
| Larynx | 55.8 (9.3–184.2) | 27.9 | 9.3 |
| Thyroid grand | 55.9 (9.4–184.5) | 27.9 | 9.4 |
| Multiple myeloma | 79.5 (13.3–262.6) | 39.7 | 13.3 |
| Central nervous system | 102.1 (17.1–337.2) | 51.0 | 17.1 |

^*a^ Same value for LL of IRR_2|1_ in principle. IRRs for each cancer type were 6.0 in this case.

Abbreviations: CI, confidence interval; IR, incidence rate; IR_2|1,_ IR of two cases in 1-year per 300 population (=2/300); IRR, incidence rate ratio LL, lower limit of 95% confidence interval.
